# Characteristics of Water Environment and Intestinal Microbial Community of Largemouth Bass (*Micropterus salmoides*) Cultured Under Biofloc Model

**DOI:** 10.3390/microorganisms12112158

**Published:** 2024-10-26

**Authors:** Yuqin Jin, Shunlong Meng, Huimin Xu, Chao Song, Limin Fan, Liping Qiu, Dandan Li

**Affiliations:** 1 Wuxi Fisheries College, Nanjing Agricultural University, Wuxi 214128, China; jinyq@stu.njau.edu.cn (Y.J.); songc@ffrc.cn (C.S.); fanlm@ffrc.cn (L.F.); 2 Freshwater Fisheries Research Center, Chinese Academy of Fishery Sciences, Risk Assessment Laboratory for Environmental Factors of Aquatic Product Quality and Safety of the Ministry of Agriculture, Key Open Laboratory of Inland Fishery Ecological Environment and Resources, Wuxi 214081, China; xuhuimin@ffrc.cn (H.X.); qiulp@ffrc.cn (L.Q.); lidandan@ffrc.cn (D.L.)

**Keywords:** Biofloc, water environment, largemouth bass, microbial communities

## Abstract

To investigate the effects of biofloc mode on the water environment and intestinal microbial community structure of largemouth bass, a 60-day culture experiment was conducted without water replacement in 300-L glass tanks. The experiment included a control group and a biofloc group, each with three replicates. The results showed the following: (i) the richness and diversity of the water environment and fish intestinal microbial community increased under the biofloc model; (ii) Proteobacteria, Patescibacteria, and Bacteroidota were the dominant phyla in the water environment of largemouth bass, while Proteobacteria, Firmicutes, Bacteroidota, Patescibacteria, and Actinobacteriota were the dominant phyla in the gut of largemouth bass. However, differences in the relative abundance and community structure of microorganisms were observed between the two groups, suggesting that the biofloc system impacts both the water environment and intestinal microbial community structure in largemouth bass culture. (iii) A correlation analysis between water quality indices and enzyme activity with microbial abundance revealed that microbial community composition could effectively reflect water quality and fish physiological health. Based on the analysis of microbial community structure, this study offers a theoretical foundation for integrating largemouth bass culture with the biofloc system, and provides valuable data for future health management and water quality control in largemouth bass production.

## 1. Introduction

According to the United Nations, the global population is expected to increase by an additional 2 billion people, reaching 9.7 billion by 2050 [1]. This presents a significant challenge for the food production industry: satisfying the escalating demand for food with the constraint of limited arable land. In this context, aquaculture is emerging as a pivotal solution, with the potential to optimize the use of diverse aquatic resources to produce a wide range of food organisms through intensive farming practices [2]. However, intensive and semi-intensive aquaculture systems have led to problems such as declining water quality, disease outbreaks, and environmental degradation, hindering the sustainability of the industry [3]. To address these challenges, biofloc technology (BFT) has emerged as an eco-friendly solution that maintains water quality, supports environmental conservation, and facilitates material cycling. BFT helps overcome key limitations in the intensive development of aquaculture, such as high stocking densities, pollution, and disease prevalence [4]. It offers a solution by stabilizing water quality without the need for water exchange, increasing the feed available to cultured organisms, enhancing microbial diversity, and reducing the proliferation of pathogenic bacteria [5,6,7]. BFT operates by adjusting the C/N ratio in water through the addition of external carbon sources, which boosts microbial activity and population [8]. These microorganisms assimilate harmful nitrogen compounds, converting them into bacterial protein, thereby regulating water quality and promoting nutrient recycling within the aquaculture system [9].

Microorganisms are important components of water bodies, participating in nutrient cycling and playing a crucial role in inhibiting pathogens [10]. The composition of the microbial community in water bodies is vital for assessing the health of aquaculture ecosystems, which has garnered increasing attention. Gut microorganisms are closely related to animal physiology, and their abundance and composition directly influence host health [11], positively affecting growth and development [12]. In recent years, researchers have used high-throughput sequencing technology to reveal the structural composition and variation patterns of microorganisms in the water environment and gut of various aquatic animals, exploring how these microorganisms contribute to water quality regulation and physiological homeostasis in different environments [13,14,15]. In summary, investigating the abundance and community structure of microorganisms has a positive effect on the healthy culture of aquatic animals.

Largemouth bass (*Micropterus salmoides*), commonly known as California bass, is noted for its low incidence of disease and tolerance to low temperatures and it is rich in proteins, calcium, and other nutrients, with levels of amino acids and vitamins significantly higher than those found in common freshwater fish, earning it the nickname “freshwater yellow croaker (*Larimichthys crocea*)” [16,17]. In recent years, with the increasing development of intensive aquaculture, extensive farming methods such as high density and high feeding rates can easily lead to water quality deterioration, fish disease occurrence, and disease spread. This has seriously impacted on the sustainable development of largemouth bass aquaculture. Currently, largemouth bass are primarily farmed in ponds, and maintaining water quality relies on frequent and substantial water changes, but this method poses problems of environmental pollution and water resource wastage [18]. So, it is imperative to find a green aquaculture model for largemouth bass. The aim is to provide a theoretical foundation for integrating largemouth bass with the biofloc culture system, offering data to support healthy farming practices and water quality management in future production. Additionally, it presents new ideas for the sustainable development of the largemouth bass aquaculture industry.

## 2. Materials and Methods

### 2.1. Experimental Design and Culture Management

This experiment was conducted at the Freshwater Fisheries Research Center of the Chinese Academy of Aquatic Sciences. A 60-day (August–October 2023) culturing experiment was conducted without water replacement in 300-L glass tanks. The experiment was set up with a control group (only feed the feed) and a biofloc group (glucose was added to maintain a C/N ratio of 15), each with three replicates. Only the amount of water lost through sampling and natural evaporation during the culture period was replenished [19]. Each glass tank was fitted with two aeration heads, positioned on opposite sides of the tank, and connected to the SHZ-D (III) circulating water multifunctional vacuum pump to enhance oxygenation and agitate the water.

The largemouth bass employed for the experiment were acquired from Jiangsu Zhongshui Dongze Agricultural Development Co., Ltd. (Wuxi, China). Following a 14-day period of culture acclimatization, robust and similarly sized largemouth bass (average weight 33.26 ± 1.18 g) were chosen for the experiment, with each tank housing a population of 20 fish. The bass were nourished with commercial puffed compound feed (purchased from Wuxi Tongwei Biotechnology Co., Ltd., Wuxi, China) and Special Materials Branch, Wuxi, China, consisting of 46.00% crude protein, 6.00% crude fiber, 6.00% crude fat, 16.00% crude ash, 1.20% total phosphorus, and 2.30% lysine, at a rate of 3% of their body weight twice a day (9:00 a.m. and 5:00 p.m.). Additionally, every 15 days, six fish were randomly selected for weighing to regulate their feed consumption. Throughout the trial, the water temperature was maintained at between 24 and 30 °C, the pH was kept within the range of 6.5–8.5, and the dissolved oxygen level was sustained at 7–9 mg/L.

Based on the C/N formula for the biofloc aquaculture system as summarized by Avnimelech [8], the amount of glucose to be added was calculated. In the experiment, the C/N ratio referred to the mass ratio of carbon to nitrogen in the added substances (feed and glucose). The amount of glucose added was adjusted according to the feed input to calculate the required amount of glucose needed to achieve a C/N ratio of 15. Glucose was added half an hour after each feed. The glucose employed for the experiment was dextrose monohydrate, sourced from Renhe Tang Pharmaceutical Co., Ltd. (Linyi, China), and it contained 36.37% carbon.

### 2.2. Sample Collection

At the 60-day mark of the culture experiment, after a 24-h fasting period for the largemouth bass, samples were procured. Two fish were randomly selected from each tank, totaling six fish from both the biofloc and control groups. Anesthesia was induced using MS-222 at a concentration of 70 mg/L. Once the fish were fully anesthetized, they were dissected to harvest their livers, intestines, and intestinal contents. These samples were then placed into 2 mL centrifuge tubes and stored in a freezer at −80 °C. Simultaneously, 1 L of water samples was collected and filtered through 0.5 μm (collecting planktonic microorganisms) and 0.02 μm (collecting attached microorganisms) mixed cellulose ester filter membranes using a filtration apparatus under negative pressure. The filtered membranes were placed into 2 mL sterile centrifuge tubes and processed alongside the intestinal content samples for subsequent DNA extraction and high-throughput sequencing experiments.

### 2.3. Microbial DNA Extraction and High-Throughput Sequencing

Genomic DNA was extracted from the samples using the E.Z.N.A.^®^ Soil DNA Kit (Omega Bio-Tek, Norcross, GA, USA), and the DNA quality was assessed via 1% agarose gel electrophoresis. Amplification of the bacterial 16S ribosomal RNA gene’s V3–V4 region was achieved through PCR, and the primers used for amplification were 341F (5′-ACTCCTACGGGAGGCAGCAG-3′) and 806R (5′-GGACTACHVGGGTWTCTAAT-3′) [20], where barcode is an eight-base sequence unique to each sample. PCR reactions were performed in triplicate 20 μL mixture containing 4 μL of 5 × FastPfu Buffer, 2 μL of 2.5 mM dNTPs, 0.8 μL of each primer (5 μM), 0.4 μL of FastPfu Polymerase, and 10 ng of template DNA. Following this, amplicons were extracted from 2% agarose gels and purified with the AxyPrep DNA Gel Extraction Kit (Axygen Biosciences, Union City, CA, USA) following the instructions given by the creator. SMRTbell libraries were prepared from the amplified DNA by blunt-ligation according to the manufacturer’s instructions (Pacific Biosciences, Menlo Park, CA, USA). Purified SMRTbell libraries from the Zymo and HMP mock communities were sequenced on dedicated PacBio Sequel II 8M cells using Sequencing Kit 2.0 chemistry. Purified SMRTbell libraries from the pooled and barcoded samples were sequenced on a single PacBio Sequel II cell. All amplicon sequencing was performed by Shanghai Biozeron Biotechnology Co., Ltd. (Shanghai, China).

The raw data were processed by trimming and filtering to obtain clean sequences. Operational taxonomic units (OTUs) were clustered with 97% similarity cutoff using UPARSE (version 7.1, http://drive5.com/uparse/ accessed on 23 January 2024), and chimeric sequences were identified and removed using UCHIME (version 4.2) [21]. The phylogenetic affiliation of each 16S rRNA gene sequence was analyzed by Ribosomal Database Project (RDP) Classifier (http://rdp.cme.msu.edu/ accessed on 23 January 2024) against the silva (SSU132) 16S rRNA database using a confidence threshold of 70% [22].

### 2.4. Water Quality and Enzyme Activity Measurement

The total nitrogen (TN), total phosphorus (TP), ammonia nitrogen (NH_4_^+^-N), nitrate nitrogen (NO_3_^−^-N), and nitrite nitrogen (NO_2_^–^-N) contents of culture water were determined by peroxide potassium sulfate-ultraviolet spectrophotometry (GB 11894-89), ammonium molybdate spectrophotometric method (GB/T 11893-89), Nessler’s Reagent Spectrophotometry (GB 7479-87), UV spectrophotometry (GB/T 5750.5-2006), and the spectrophotometric method (GB 7493-87).

The liver and intestinal samples were blended with precooled phosphate-buffered saline at a pH of 7.2 to 7.4 in a ratio of 1 g of tissue to 9 milliliters of buffer. Following this, the samples were homogenized to a fine consistency. The prepared mixture was then placed in a refrigerated centrifuge and spun at 12,000× *g* at a chilled temperature of 4 °C for a duration of 10 min. After centrifugation, 2 milliliters of the supernatant were extracted for subsequent analysis. The activities of intestinal amylase (AL), intestinal lipase (LPS), liver superoxide dismutase (SOD), liver catalase (CAT), liver alkaline phosphatase (AKP), and liver acid phosphatase (ACP) were quantitatively evaluated using protocols provided with commercial assay kits (Shanghai Enzyme Link Biotechnology Co., Ltd. Shanghai, China). The levels of soluble proteins in all enzyme extracts were measured using Bradford’s method [23]. Each enzyme assay was performed in triplicate within a 96-well microplate format and executed at a temperature of 25 °C with the MicroStation Identification System (Biolog Inc., Hayward, CA, USA).

### 2.5. Statistical Analysis

The collected parameters underwent a thorough analysis and were organized within Excel 2021. The findings from the comprehensive data analysis were reported in the format of mean ± standard deviation (Mean ± SD). The normality of all data was assessed with the Shapiro–Wilk test, and the homogeneity of variances was investigated using Levene’s test. Following this, an independent samples t-test was utilized to facilitate a comparative analysis between the control and biofloc groups. The statistical evaluation was performed with the aid of SPSS Statistics version 27.0 software (SPSS Inc., Chicago, IL, USA), where a *p*-value below 0.05 was considered to indicate statistical significance. Microbial species composition analysis, diversity analysis, principal component analysis (PCoA), and correlation analysis were done through the cloud platform of Shanghai Biozeron Biotechnology Co., Ltd. (Shanghai, China). In this, non-parametric multivariate analysis of variance (PERMANOVA) was employed to determine significant differences in microbial community composition, and the Spearman rank correlation was used to investigate the relationships between environmental factors and microbial species abundance, yielding pairwise correlations and their statistical significance.

## 3. Results

### 3.1. Microbial Diversity

#### 3.1.1. OUT Clustering, Species Annotation, and Rarefaction Curves

**Figure 1:**
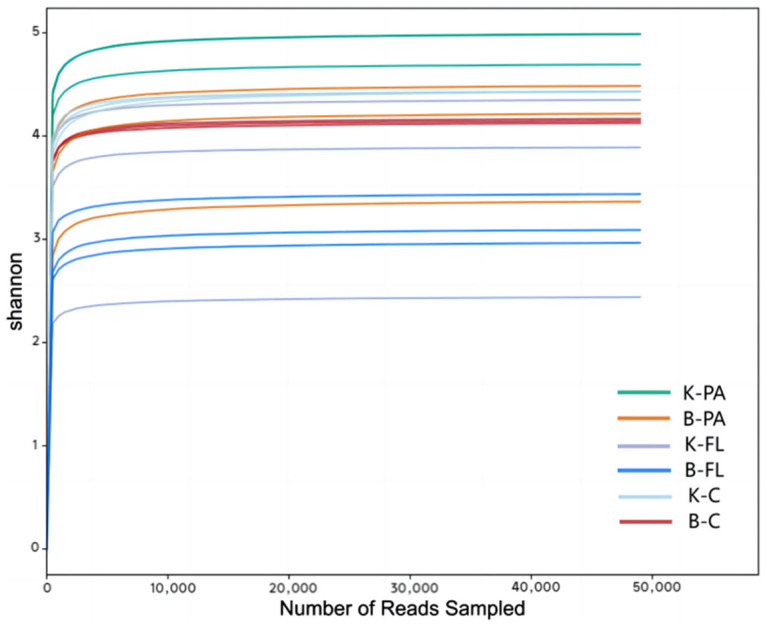
Rarefaction curves for different sample groups. Note: K-PA: attached microorganisms in the water in the control group, B-PA: attached microorganisms in the water in the biofloc group, K-FL: planktonic microorganisms in the water in the control group, B-FL: planktonic microorganisms in the water in the biofloc group, K-C: gut microorganisms of largemouth bass in the control group, B-C: gut microorganisms of largemouth bass in the biofloc group; the same below.

High-throughput sequencing was performed on water and intestinal samples collected during the experiment. The RDP classifier Bayesian algorithm was used for taxonomic analysis of the representative sequences of OTUs at a 97% similarity level. Species annotation was conducted by comparing the representative OTU sequences with a reference database. In total, 44 phyla, 183 classes, 247 orders, 344 families, 646 genera, and 201 species were identified. The statistical analysis at different classification levels revealed a total of 11,545 OTUs, with 100.00% annotated at the phylum level, 98.33% at the class level, 94.03% at the order level, 76.83% at the family level, 52.90% at the genus level, and 5.40% at the species level. Rarefaction curves can explain the differences in species richness, determine the required sample size for testing samples, and confirm the validity of the data. The rarefaction curve analysis of each group of samples (Figure 1) indicated that the curves eventually flattened out, suggesting that, as more sequencing data were obtained, the number of OTU types decreased. This trend indicates that the sequencing results are reasonable, with a flatter curve reflecting the diversity information of most species in the sample more accurately.

#### 3.1.2. Alpha Diversity Analysis of Microbial Communities

To investigate the changes in microbial communities in the culture system and fish gut during the trial period, high-throughput sequencing technology was used to assess the alpha diversity of both attached and planktonic microorganisms in the water and intestinal samples of largemouth bass from the control group and the biofloc groups. Alpha diversity indices, including Observed species, Chao1, ACE, Shannon, Simpson, Pielou_J, and Pd_faith, were calculated using Qiime software (version 1.8.0). Among these, Observed species, Chao1, and ACE represent richness metrics, Shannon and Simpson indicate diversity, Pielou_J measures evenness, and Pd_faith reflects evolutionary diversity.

The alpha diversity indices for attached and planktonic microorganisms in the water, as well as for the gut microbial communities in both the control and biofloc groups, were shown in Table 1. The Observed species, Chao1, ACE, and Pd_faith indices of attached and planktonic microorganisms were higher in the biofloc group than in the control group, with a statistically significant difference noted only for the Pd_faith index (*p* < 0.05). However, the Shannon, Simpson, and Pielou_J indices were lower in the biofloc group compared to the control group.

For the gut microbial community, all alpha diversity indices (Observed species, Chao1, ACE, Shannon, Simpson, Pielou_J, and Pd_faith) were higher in the biofloc group, with a significant difference observed only for the Shannon index (*p* < 0.05). These findings indicate that the biofloc culture system led to increased microbial richness and diversity in both the water and fish gut to some extent.

**Figure 2:**
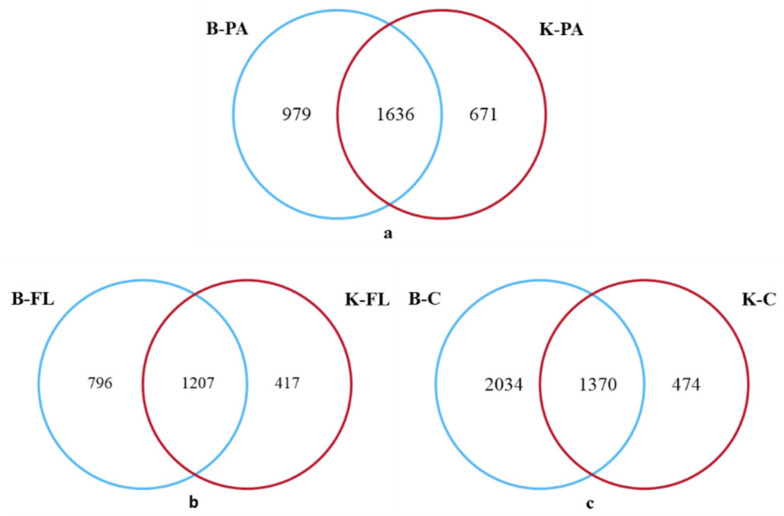
(**a**–**c**) Venn diagram of OUT number for different samples.

According to the Venn diagram, 1636 OTUs of attached microorganisms were shared between the biofloc and control groups, with 979 unique OTUs in the biofloc group and 671 in the control group (Figure 2a). For planktonic microorganisms, 1207 OTUs were shared between the groups, while 796 unique OTUs were found in the biofloc group and 417 in the control group (Figure 2b). In the gut microorganisms of largemouth bass, 1370 OUTs were shared between the two groups, with 2034 unique OTUs in the biofloc group and 474 in the control group (Figure 2c). It is evident that under the biofloc model, the unique OTUs in both the water environment and the gut of largemouth bass increased, which positively contributes to enhancing microbial diversity.

### 3.2. Microbial Community Composition

#### 3.2.1. Community Composition

**Figure 3:**
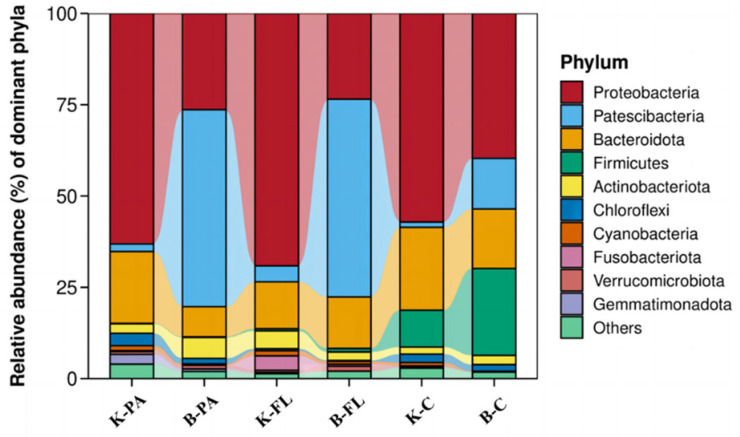
Community composition at phylum classification level in different samples.

As shown in Figure 3, at the end of the culture period, the proportions of Proteobacteria in the attached microorganisms in the water of the biofloc group and the control group were 26.36% and 63.11%, respectively. The proportions of Patescibacteria were 53.44% and 2.10%, respectively, while Bacteroidota accounted for 8.25% and 21.67%, respectively. Additionally, the proportions of Actinobacteriota were 4.74% in the biofloc group and 1.60% in the control group, while Chloroflexi made up 3.04% and 2.40%, respectively. At the phylum level, the dominant groups of attached microorganisms in the biofloc group’s water were Patescibacteria, Proteobacteria, Bacteroidota, and Actinobacteriota, whereas in the control group, the dominant groups were Proteobacteria, Bacteroidota, and Chloroflexi. These dominant phyla constituted 94.29% of the total microbial community in the biofloc group and 86.17% in the control group, highlighting their significant positions in the community structure. Furthermore, while the dominant microbial groups were similar between the two groups, the relative abundances of each phylum varied considerably.

As shown in Figure 3, at the end of the culture, the relative abundances of Proteobacteria in the planktonic microorganisms in the water of the biofloc group and the control group were 23.50% and 69.13%, respectively. The proportions of Patescibacteria were 54.13% and 4.36%, respectively, while Bacteroidota accounted for 14.12% and 12.85%, respectively. Collectively, these three dominant phyla constituted 91.75% of the total planktonic microbial community in the biofloc group and 86.17% in the control group, underscoring their prominent roles within the microbial composition. Compared to the control group, the biofloc group exhibited a lower relative abundance of Proteobacteria but higher relative abundances of Patescibacteria and Bacteroidota. This indicates that, at the phylum level, while the compositions of the dominant phyla in the planktonic microbial communities of both groups were similar, the relative abundances of each phylum differed between the two groups.

As shown in Figure 3, at the phylum level, the dominant bacterial groups in the intestines of largemouth bass in the biofloc group were Proteobacteria (39.65%), Firmicutes (23.81%), Bacteroidota (16.28%), Patescibacteria (13.91%), and Actinobacteriota (2.53%). The combined relative of these five phyla exceeded 90%. The composition of dominant bacterial groups in the intestinal microbiota of largemouth bass in the control group was similar, but the relative abundances of each phylum differed. In the control group, the proportions of these phyla were 57.13%, 10.10%, 22.72%, 1.42%, and 1.93%, respectively, with a total relative abundance exceeding 90%. Notably, the relative abundances of Firmicutes, Patescibacteria, and Actinobacteriota were higher in the biofloc group compared to the control group, while the relative abundances of Proteobacteria and Bacteroidota were lower. These differences suggest that the biofloc system had a significant influence on the composition of the intestinal microbial community structure of largemouth bass. Notably, the relative abundance of Patescibacteria in both the water environment and the fish intestines in the biofloc group was significantly higher (*p* < 0.05) than in the control group. This suggests that the biofloc environment may promote the proliferation of this phylum. The increased abundance of Patescibacteria could indicate more efficient nutrient cycling, improved water quality management, and a healthier ecological balance within the biofloc system, which holds positive implications for practical aquaculture applications

#### 3.2.2. Similarities and Differences in Microbial Communities

**Figure 4:**
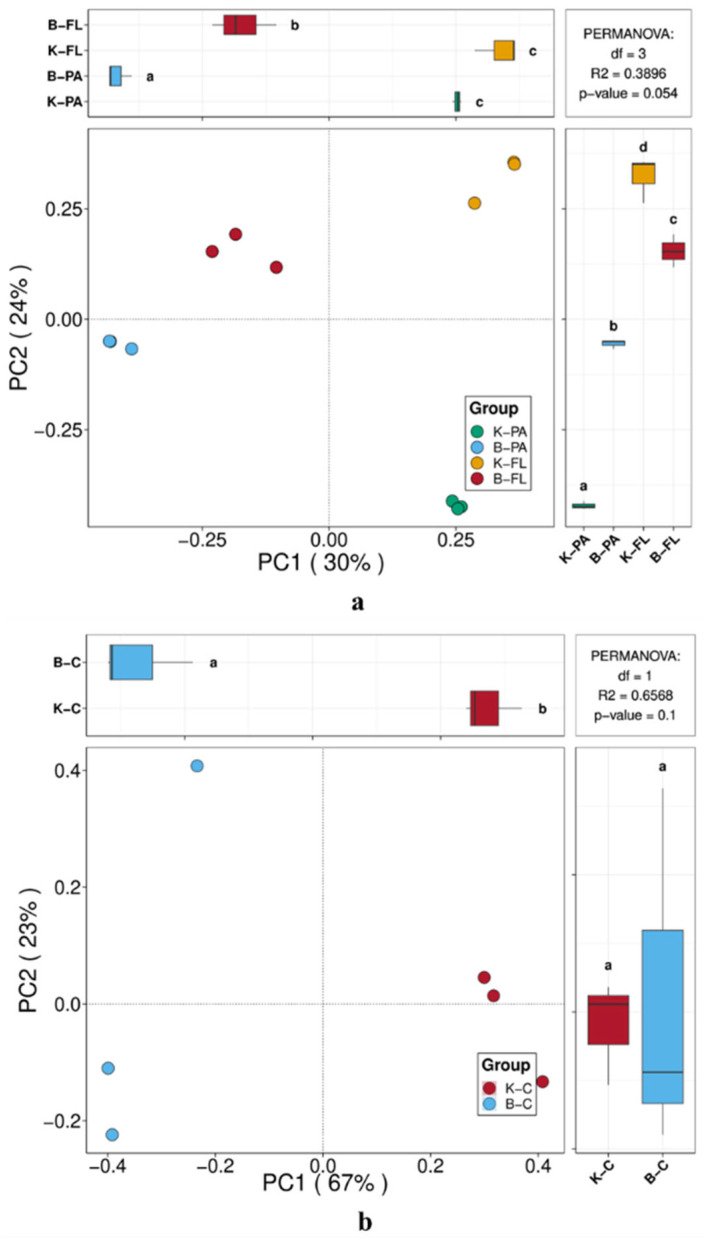
(**a**,**b**) PCoA analysis different samples based on Bray–Curtis distance. Note: the lowercase letters above and beside the boxes represent the significance test results of beta diversity distance differences between samples. Different letters indicate that the *p*-value of the difference test between groups is less than 0.05.

At the end of the culture, the PCoA analysis of attached and planktonic microorganisms in the water of the two culture systems were shown in Figure 4a. PCoA1 accounted for 30% of the variation on the horizontal axis, and PCoA2 explained 24% of the variation on the vertical axis. The attached microbial communities from the biofloc group and the control group clustered separately in the third and fourth quadrants, respectively. According to the beta diversity distance significance test, there was a significant difference in the structure attached microbial communities between the biofloc group and the control group (*p* < 0.05). Similarly, planktonic microbial communities from the biofloc and control groups clustered in the second and first quadrants, respectively, also showing a significant difference (*p* < 0.05). These results indicate that the biofloc system led to significant changes in the microbial community structure of the water environment.

Figure 4b showed the PCoA analysis of the intestinal microorganisms of largemouth bass from the biofloc and control groups. PCoA1 explained 67% of the variation on the horizontal axis, while PCoA2 accounted for 23% on the vertical axis, together representing 90% of the variation. This suggests that these two principal components effectively reflect the intestinal microbial diversity in the culture systems. The intestinal microbial samples from the biofloc group clustered in the second and third quadrants, while those from the control group clustered in the first and fourth quadrants, with a significant difference between the groups (*p* < 0.05). These findings further demonstrate the impact of the biofloc culture system on the intestinal microbial community structure of largemouth bass.

### 3.3. Environmental and Physiological Factor Correlation Analysis

#### 3.3.1. Differences in Water Quality

A comparison of some water quality indicators between the biofloc group and the control group at the end of the culture was shown in Table 2. The contents of ammonia nitrogen, nitrite nitrogen, nitrate nitrogen, total nitrogen, and total phosphorus in the water of the biofloc group were significantly lower than those of the control group (*p* < 0.05). Specifically, at the end of culture, the ammonia nitrogen, nitrite nitrogen, nitrate nitrogen, total nitrogen, and total phosphorus contents in the biofloc group were reduced by 57.07%, 80.22%, 30.50%, 24.64%, and 31.47%, respectively, compared to the control group.

#### 3.3.2. Correlation of Water Microorganisms with Water Quality Indicators

**Figure 5:**
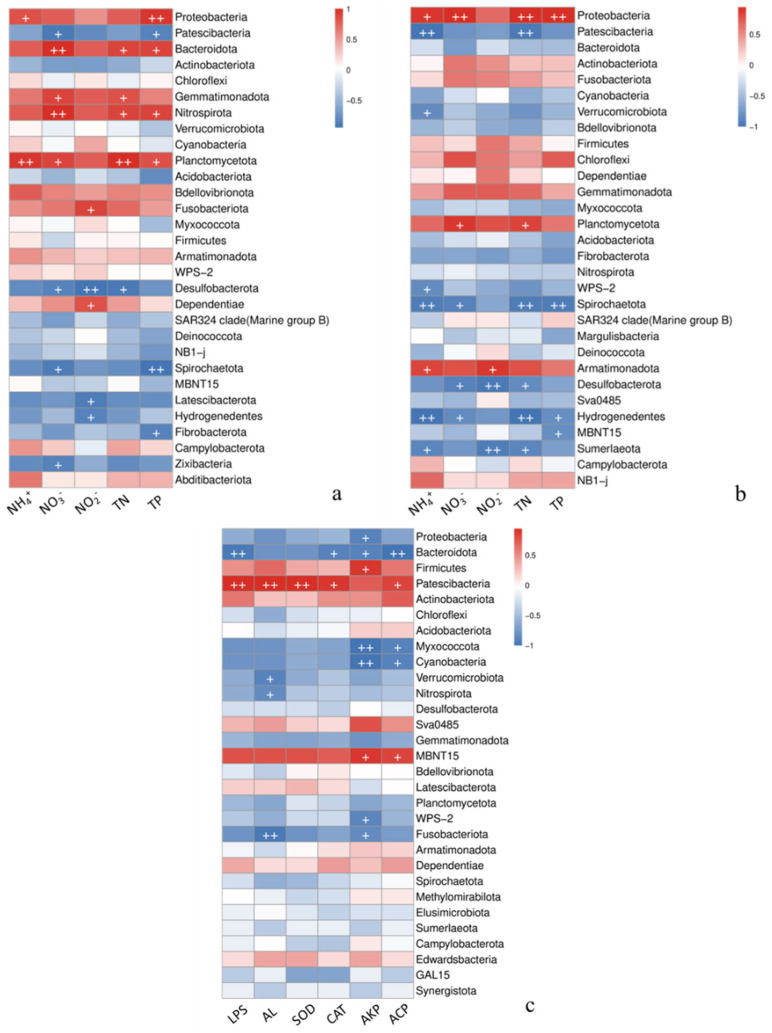
(**a**,**b**) Correlation analysis between the abundance of major species and environmental factors at the phylum level. (**c**) Correlation analysis between the abundance of major species and physiological factors at the phylum level. Note: “+” indicates that the *p*-value of the significance test of correlation analysis between the abundance of major species and environmental factors at the phylum level is less than 0.05. “++” indicates that the *p*-value l is less than 0.01; the same below.

Through Heatmap correlation analysis, environmental factors and the levels of attached and planktonic microorganisms in the water were clustered by averaging. The top 30 species in terms of abundance at the phylum level were selected for Spearman’s rank correlation calculation. For attached microorganisms (Figure 5a), environmental factors such as nitrate nitrogen, nitrite nitrogen, total nitrogen, and total phosphorus had a stronger impact on microbial communities than ammonia nitrogen. In terms of microbial phyla, ammonia nitrogen showed a significant positive correlation with Proteobacteria and Planctomycetota (*p* < 0.05). Nitrate nitrogen, nitrite nitrogen, total nitrogen, and total phosphorus were positively correlated with Bacteroidota, Gemmatimonadota, Nitrospirota, and Planctomycetota, indicating that these four phyla had similar requirements for environmental factors, with nitrate nitrogen, total nitrogen, and total phosphorus exhibiting significant correlations with them (*p* < 0.05). Conversely, ammonia nitrogen, nitrate nitrogen, nitrite nitrogen, total nitrogen, and total phosphorus were negatively correlated with Patescibacteria. Only nitrate nitrogen and total phosphorus showed significant correlations with Patescibacteria (*p* < 0.05). Patescibacteria exhibited opposite correlations to the aforementioned phyla, suggesting potential antagonistic or competitive relationships with other phyla.

Figure 5b showed that the phyla most strongly correlated with environmental factors among the planktonic microorganisms in the water were Proteobacteria, Spirochaetota, and Hydrogenedentes, all of which exhibited significant correlations with the four types of environmental factors (*p* < 0.05). Notably, Planctomycetota exhibited a significant positive correlation with nitrate nitrogen and total nitrogen (*p* < 0.05). Conversely, Desulfobacterota showed a significant negative correlation with nitrate nitrogen and total nitrogen (*p* < 0.05), indicating that these two phyla exhibit similar sensitivity to changes in nitrate nitrogen and total nitrogen, and there was a possibility of a competitive relationship. These findings indicate that fluctuations in environmental factors substantially impact the stability of microbial communities in the water, rapidly altering their composition and function.

#### 3.3.3. Differences in Enzyme Activity of Largemouth Bass

The enzyme activity indices of largemouth bass in the biofloc and control groups at the end of the culture period were shown in Table 3. At the end of culture, the activities of LPS, AL, SOD, CAT, AKP, and ACP of largemouth bass in the biofloc group were significantly higher than those of the control group (*p* < 0.05). Specifically, the enzyme activities in the biofloc group increased by 52.79%, 35.29%, 51.16%, 47.41%, 39.35%, and 29.80%, respectively, compared to the control group.

#### 3.3.4. Correlation of Intestinal Microorganisms with Enzyme Activity

Heatmap correlation analysis combined with Spearman’s rank correlation was used to investigate the interrelationships between environmental factors and the top 30 most abundant species in the intestinal microbiota of largemouth bass, yielding pairwise correlations. As shown in Figure 5c, the correlation between the intestinal microbiota and environmental factors was lower than that of the water microbiota. Proteobacteria and Bacteroidota showed negative correlations with LPS, AL, SOD, CAT, AKP, and ACP. Specifically, Proteobacteria had a significant negative correlation with AKP (*p* < 0.05), while Bacteroidota showed significant negative correlations with LPS, CAT, AKP, and ACP (*p* < 0.05). In contrast, Firmicutes and Patescibacteria exhibited positive correlations with these six environmental factors. Notably, Firmicutes had a significant positive correlation with AKP (*p* < 0.05), and Patescibacteria displayed significant positive correlations with LPS, AL, SOD, CAT, and ACP (*p* < 0.05). These findings suggest that Firmicutes and Patescibacteria have different environmental factors requirements compared to Proteobacteria and Bacteroidota, implying the coexistence of microbiota with opposite functional roles in the intestine, working synergistically to maintain intestinal homeostasis.

## 4. Discussion

The rise of intensive aquaculture models has significantly contributed to the growth of the aquaculture industry. However, these systems have altered the natural material cycling and energy flow within farming environments, often resulting in the gradual degradation of water quality. The species and richness of microbial communities in water serve as key indicators of water quality. Ma et al. [24] have reported that higher microbial diversity leads to greater resistance to environmental changes. They also emphasized that the biofloc culture system can help maintain ecosystem stability under minor environmental fluctuations. In this study, the biofloc culture mode for largemouth bass was found to regulate the diversity of the microbial community in the water, increasing both diversity and abundance, while also improving community evenness and maintaining structural stability. This is consistent with numerous studies confirming that microbial communities in freshwater apuaculture systems are primarily composed of phyla such as Actinobacteriota, Verrucomicrobiota, Planctomycetes, Cyanobacteria, Patescibacteria, Proteobacteria, and Bacteroidota [25]. Other studies have observed significant shifts in microbial community structure during the initial stages of biofloc system cultivation. However, as the system matures and stabilizes, groups such as Proteobacteria, Planctomycetes, Cyanobacteria, Bacteroidota, and Firmicutes often become the dominant microbial communities [26]. In this experiment, in both biofloc and the control groups, the dominant bacterial phyla were Proteobacteria, Patescibacteria, Bacteroidota, and Actinobacteria, corroborating previous reports. Interestingly, in the biofloc group, Patescibacteria accounted for over 50% of both attached and planktonic microorganisms, indicating that it was the dominant phylum in the biofloc system. Patescibacteria, which are known to form epibionts on Actinobacteria, constitute a significant proportion of microbial “dark matter” and may contain valuable biochemical pathways with potential biotechnology applications [27]. These microorganisms offer insights into microbial ecosystem functioning and the cell biology of various species within biofloc systems. In addition to Patescibacteria, Proteobacteria also dominated both attached and planktonic microorganisms in the biofloc group, mirroring findings by Deng et al. [28], although the relative abundance of Proteobacteria was lower in our study. Other studies have reported that Proteobacteria constituted 58.23% and 49.88% of the microbial community in the biofloc-cultured water [29], with variations likely due to differences in carbon source types and feeding strategies. Chloroflexi was more abundant in attached microorganisms in the biofloc group than in the control group. This can be attributed to the fact that Chloroflexi contains various filamentous bacteria that play a key structural role in biofloc formation [30], and the different cultivation modes led to this variation. Similar to previous studies, Chloroflexi was found to support the stability of the bacterial-algal symbiotic biofilms [31]. The biofloc group required additional glucose based on daily feed intake, whereas the control group did not receive any additional carbon source. This likely explains the higher abundance of Actinobacteriota in the biofloc group, as these microorganisms are known to degrade complex carbohydrates [32]. Among the planktonic microorganisms, in addition to Patescibacteria and Proteobacteria, Bacteroidota was also a dominant bacterial phylum in the biofloc group, with a higher abundance than in the control group. Consistent with previous findings, Bacteroidota was found to relatively abundant under the biofloc mode [33], further supporting the results of this study. This may suggest that Bacteroidota plays a crucial role in organic matter degradation and nutrient cycling within the biofloc system. It also indicates the importance of this microbial community in maintaining water quality, promoting system stability, and contributing to the functional differentiation of the ecosystem.

Ammonia nitrogen, nitrate nitrogen, nitrite nitrogen, total nitrogen, and total phosphorus are widely used physicochemical parameters for assessing water quality, as they collectively influence the aquatic environment [34]. Excessive nutrient enrichment, particularly of nitrogen and phosphorus, can trigger the rapid proliferation of algae or aquatic plants, resulting in harmful algal blooms and subsequent water quality degradation [35]. In addition, increased levels of ammonia nitrogen and nitrite nitrogen pose direct health risks to aquatic animals [36], making it crucial to regulate nitrogen and phosphorus concentrations in systems. Moreover, the physicochemical factors of water are closely linked to the composition of microbial communities [37]. In this study, the concentrations of ammonia nitrogen, nitrate nitrogen, and total phosphorus were significantly lower in the biofloc group compared to the control group. This reduction may be attributed to the distinct microbial community structures in the water. Specifically, in the biofloc group, the relative abundances of Patescibacteria, Actinobacteriota, and Chloroflexi were higher among the attached microorganisms, while Bacteroidota was more abundant among planktonic microorganisms. These microbial groups play key roles in the degradation of inorganic compounds [25], likely contributing to the observed differences in water quality between the two groups. The results demonstrated a strong correlation between the microbial community structure and the physicochemical parameters of the water. Specifically, among the attached and planktonic microorganisms, Proteobacteria showed a significant positive correlation with ammonia nitrogen and total phosphorus content. This suggests that, as the levels of these nutrients increase, the abundance of Proteobacteria also rises, which is consistent with their role in promoting nitrification, facilitating the conversion of ammonia nitrogen into nitrite nitrogen and nitrate nitrogen [38]. Theoretically, a higher abundance of Proteobacteria should correlate with lower levels of ammonia nitrogen and nitrite nitrogen. However, in this experiment, the control group exhibited higher concentrations of these nitrogenous compounds than the biofloc group. The likely explanation is that nitrification in the biofloc system was driven by the synergistic activity of autotrophic and heterotrophic bacteria. The daily supplementation of a carbon source in the biofloc group provided an essential substrate for heterotrophic nitrifying bacteria, promoting the rapid growth of nitrifying microorganisms, including Actinomycetes within *Actinomycetes* [39]. This also explains the higher abundance of Actinobacteriota in the biofloc group compared to the control group. Conversely, Patescibacteria exhibited a negative correlation with ammonia nitrogen, nitrate nitrogen, nitrite nitrogen, and total phosphorus in both attached and planktonic microorganisms, suggesting that Patescibacteria plays a positive role in nitrogen and phosphorus removal from the water. Previous studies have indicated that Patescibacteria can utilize nitrate nitrogen to facilitate nitrification, thereby reducing ammonia nitrogen and nitrite nitrogen concentrations, while also contributing to the removal of total phosphorus [40]. These findings are consistent with the results of this study, further validating the complex relationship between environmental factors and aquatic microbial communities. In conclusion, by analyzing the correlations between environmental parameters and the structure of aquatic microbial communities, water quality can be better understood and managed. This approach provides valuable insights into optimizing aquaculture practices and improving overall culture system efficiency.

The intestinal microbiota of fish is diverse and plays a crucial role in maintaining overall homeostasis. Meanwhile, research on these microbial communities is significant for elucidating fish immune responses, pathogen invasion processes, growth, and development [41]. According to the diversity index results, the Chao1, ACE, Shannon, and other indices were higher in the biofloc group compared to the control group, indicating that the biofloc system increased species’ richness and diversity in the intestinal microbiota of largemouth bass, thereby contributing to the stability of the intestinal environment. Dong et al. [42] used a biofloc system to culture Pacific white shrimp (*Penaeus vannamei*) and found that the species richness and diversity of the shrimp’s intestinal microbiota increased, leading to improve the shrimp’s survival rate. Huang et al. [43] found that the alpha diversity of the intestinal microbiota of tilapia (*Oreochromis mossambicus*) increased under the biofloc mode, which positively impacted intestinal health. The results of the present study align with these findings. Different fish species exhibit unique intestinal microbiota structures, with Firmicutes, Bacteroidota, and Proteobacteria being commonly dominant microbiota. Studies have shown that the dominant microbiota in the intestine of largemouth bass are Firmicutes and Proteobacteria [44]. Biofloc systems, particularly those utilizing biodegradable polymer carbohydrates, have also demonstrated that Proteobacteria, Bacteroidota, and Firmicutes are dominant in fish intestinal microbiota [45], consistent with the results of this study. The structure of gut microbial communities is one of the important factors influencing microbial function and fish health. In this study, while the dominant microbial phyla in the intestines of largemouth bass were similar between the biofloc and control groups, differences were observed in their relative abundances. This is likely due to the biofloc system, where microorganisms in the bioflocs, ingested by the fish, colonize the gut and serve as additional nutrients [46]. In our study, Proteobacteria, which dominated the gut microbiota in both groups at the phylum level, are also abundant in natural waters and account for more than 40% of the freshwater bacterial biomass [47]. Many microorganisms within the Firmicutes phylum are known for their ability to convert carbohydrates, aiding in nutrient utilization by the host [48]. Both Bacteroidota and Firmicutes are major gut microbiota and share a symbiotic relationship, jointly facilitating nutrient absorption or energy storage in the host. Furthermore, a higher Firmicutes/Bacteroidota ratio is associated with better fish health, growth, and development [48,49]. In this study, the relative abundance of Firmicutes in the biofloc group was higher than that in the control group, while Bacteroidota was lower in the biofloc group. This indicates that the biofloc mode supports healthier growth in largemouth bass. In addition, the relative abundances of Patescibacteria and Actinobacteriota in the biofloc group were also higher than those in the control group. They not only promote biofloc formation but also provide secondary protection against fish pathogens [31,50]. Previous studies have highlighted that changes in the aquatic environment can influence the composition and stability of gut microbiota, and the formation of gut microbiota is closely related to environmental microorganisms [51]. Interestingly, while Patescibacteria had a higher relative abundance in the water of the biofloc group, its proportion in the gut of largemouth bass was lower. Conversely, Firmicutes, which had a lower relative abundance in the water of both groups, exhibited a higher relative abundance in the fish gut. This is consistent with Liu et al., who found that only 12.69% of the gut microbiota in largemouth bass originated from the aquatic environment, and some dominant genera in the water were absent in the fish gut [13]. This observation aligns with the findings of the present study. The possible reason is that the fish’s intestinal environment differs significantly from the aquatic environment, with the intestine providing unique nutritional conditions and physicochemical characteristics (such as low oxygen levels and rich organic matter). Patescibacteria may have adapted to the specific ecological niche within the fish intestine, allowing them to colonize and proliferate more effectively in this environment. This also suggests that they may possess specific ecological functions, particularly those related to the host. Such functional differentiation highlights the distinct roles of microbial communities in different ecological niches (e.g., aquatic versus intestinal environments), reflecting the complex dynamics of microbial communities in supporting aquaculture ecosystems.

The enzyme activity indicators such as LPS, AL, and SOD in the biofloc group and the control group of largemouth bass fluctuated correspondingly as the culture experiment progressed, likely due to their close relationship with the composition and structure of intestinal microbial communities. This study revealed that Proteobacteria, Firmicutes, and Bacteroidota were among the most abundant bacterial phyla in the fish intestine, and these bacterial groups were strongly associated with carbohydrate and protein degradation, inflammation reduction, and immune enhancement—functions that contribute to intestinal homeostasis and overall health [52]. The results of this experiment indicated that there was a certain correlation between the enzyme activities of LPS, AL, SOD, CAT, AKP, and ACP in largemouth bass and the composition of their intestinal microbial communities. Specifically, Proteobacteria showed a negative correlation with these enzyme activities, suggesting that an excessive proportion of Proteobacteria in the gut might hinder the normal growth and health of the organism [53]. For instance, most intestinal pathogenic bacteria belong to Proteobacteria, such as Escherichia (*Escherichia Castellani and Chalmers, 1919*), *Salmonella*, *Klebsiella*, *Shigella Castellani*, *Yersinia*, *Pseudomonas*, and *Vibrio cholerae*. This supports the viewpoint proposed by some researchers that “an increase in Proteobacteria abundance is a potential risk for disease” [54,55]. In contrast, Firmicutes showed a positive correlation with these enzyme activities, particularly a significant positive correlation with AKP, indicating that Firmicutes play an active role in regulating intestinal microbiota balance and promoting metabolic processes. This supports the classification of Firmicutes as a probiotic phylum [29,56]. The beneficial effects of Firmicutes are often linked to their metabolites, which include various biologically active compounds. For example, members of the genus *Bacillus* (within Firmicutes) produce numerous useful enzymes and antibacterial compounds, some of which may have antibacterial, antiviral, or antifungal properties [57,58]. Under the experimental conditions, the proportions of Proteobacteria and Firmicutes, as the dominant intestinal microorganisms, varied between the biofloc and control groups, which contributed to the observed differences in enzyme activities. This also explains why the largemouth bass in biofloc group demonstrated improved digestive performance, antioxidant capacity, and immune response. Bacteroidota, on the other hand, showed a significant negative correlation with the activities of LPS, CAT, AKP, and ACP. This indicates that Bacteroidota may suppress the activity of certain digestive, antioxidant, and immune enzymes, thereby negatively impacting the physiological health of the fish. Previous studies have associated an increase in Bacteroidota abundance with inflammatory bowel disease (IBD), an intestinal disorder [56]. Additionally, Bacteroidota infections can have detrimental effects on fish health, as these bacteria are capable of degrading complex biopolymers (polysaccharides, proteins and so on), which are known virulence factors [59]. This supports the earlier observation that a higher Firmicutes/Bacteroidota ratio is more beneficial for fish health, which may explain why the largemouth bass in the biofloc group exhibited better physiological conditions, whereas Patescibacteria showed a positive correlation with most of the enzyme activities, except AKP, with significant positive correlations with LPS, AL, SOD, CAT, and ACP. This indicates that Patescibacteria plays a beneficial role in supporting fish physiological health, though further research is needed to clarify the specific mechanisms by which this phylum regulates these processes. In conclusion, analyzing the correlation between the abundance of intestinal microbial communities and enzyme activities provides valuable insights into changes in the intestinal environment and physiological health status of fish. This understanding can be applied to promote healthier aquaculture practices and ensure the well-being of cultured fish.

## 5. Conclusions

In conclusion, our results showed that (i) the richness and diversity of culture water environment and fish intestinal microbial community increased to a certain extent under the biofloc model. (ii) Proteobacteria, Patescibacteria, and Bacteroidota were the dominant phyla in the culture water environment of largemouth bass, while Proteobacteria, Firmicutes, Bacteroidota, Patescibacteria, and Actinobacteriota were the dominant phyla in the gut of largemouth bass. However, there were differences in the relative abundance and community structure of microorganisms between the two groups. It can be seen that biofloc model of largemouth bass culture has an impact on the culture water environment and intestinal microbial community structure. (iii) The correlation analysis of water quality indexes and enzyme activity indexes with the abundance of microbial dominant phyla revealed that the abundance of the microbial community could effectively reflect the water quality and the physiological health of fish. This study highlights the significance of the biofloc model in aquaculture by demonstrating its positive impact on both the water environment and the intestinal microbial community of largemouth bass, while also revealing that the abundance and structure of microbial communities serve as valuable indicators for monitoring water quality and fish health, offering important insights for the sustainable development of aquaculture.

## Figures and Tables

**Table 1:** Alpha diversity index of different samples.

Groups	Observed_Species	Chao1	ACE	Shannon	Simpson	Pielou_J	Pd_Faith
K-PA	1581.67 ± 162.86	2079.39 ± 203.95	2130.30 ± 232.19	4.88 ± 0.17	0.97 ± 0.00	0.66 ± 0.01	63.60 ± 6.93 ^a^
B-PA	1741.67 ± 38.37	2264.67 ± 141.11	2346.52 ± 102.62	4.01 ± 0.59	0.84 ± 0.12	0.54 ± 0.08	79.31 ± 3.69 ^b^
K-FL	1075.33 ± 139.27	1477.97 ± 207.24	1550.91 ± 196.43	3.55 ± 1.00	0.87 ± 0.12	0.51 ± 0.14	54.92 ± 5.16 ^a^
B-FL	1335.00 ± 43.31	1893.03 ± 119.74	1977.77 ± 95.64	3.14 ± 0.25	0.79 ± 0.07	0.44 ± 0.04	72.16 ± 2.88 ^b^
K-C	1098.67 ± 24.17	1542.65 ± 67.74	1646.48 ± 28.66	4.07 ± 0.03 ^a^	0.93 ± 0.02	0.58 ± 0.00	55.20 ± 2.41
B-C	1619.67 ± 359.90	1764.22 ± 227.02	1865.37 ± 190.44	4.39 ± 0.04 ^b^	0.94 ± 0.00	0.60 ± 0.02	84.50 ± 25.74

Note: Different letters in the same column in the table are significant difference (*p* < 0.05).

**Table 2:** Differences in water quality indicators in largemouth bass culture systems between biofloc and control groups.

Index	Control Group	Biofloc Group
Ammonia nitrogen content (mg/L)	1.98 ± 0.09 ^a^	0.85 ± 0.05 ^b^
Nitrous nitrogen content (mg/L)	0.91 ± 0.02 ^a^	0.18 ± 0.01 ^b^
Nitrate nitrogen content (mg/L)	50.16 ± 0.66 ^a^	34.86 ± 0.49 ^b^
Total nitrogen content (mg/L)	59.71 ± 5.99 ^a^	45.88 ± 1.47 ^b^
Total phosphorus content (mg/L)	16.62 ± 1.15 ^a^	11.39 ± 0.40 ^b^

Note: different letters in the same row in the table are significant difference (*p* < 0.05); the same below.

**Table 3:** Differences in enzyme activity of largemouth bass between biofloc and control groups.

Index	Control Group	Biofloc Group
LPS activity (umol/min/mg prot)	0.269 ± 0.009 ^a^	0.411 ± 0.003 ^b^
AL activity(mg/min/mg prot)	0.085 ± 0.001 ^a^	0.115 ± 0.001 ^b^
SOD activity (U/mg prot)	31.230 ± 0.447 ^a^	49.706 ± 0.700 ^b^
CAT activity (U/mg prot)	18.023 ± 0.147 ^a^	26.568 ± 0.229 ^b^
AKP activity (IU/g prot)	2.005 ± 0.012 ^a^	2.794 ± 0.010 ^b^
ACP activity (IU/g prot)	1.564 ± 0.002 ^a^	2.030 ± 0.003 ^b^

Note: different letters in the same row in the table are significant difference (*p* < 0.05); the same below.

## Data Availability

All datasets generated for this study are available.
